# Left precuneus ALFF is associated with arterial oxygenation and cognitive impairment in patients with COPD: a resting-state fMRI study

**DOI:** 10.3389/fneur.2026.1906087

**Published:** 2026-08-19

**Authors:** Hongxia Bai, Sishi Zhu, Long Chi, Zhonghua Wang, Jing Shen, Xiajun Wu, Weiying Chen, Ying Bai, Yangli Yu, Qiongying Xu, Jueyue Yan, Lingli Lou

**Affiliations:** 1 Department of Critical Care Medicine, Hangzhou Third People’s Hospital (Hangzhou Third Hospital Affiliated to Zhejiang Chinese Medical University), Hangzhou, Zhejiang, China; 2 Pingyang County Hospital of Traditional Chinese Medicine, Wenzhou, Zhejiang, China; 3 Intensive Care Unit, The First People’s Hospital Of Yuhang District, Hangzhou, China; 4 Department of Emergency, The First People’s Hospital of Yuhang District, Hangzhou, China; 5 Department of Critical Care Medicine, The First Affiliated Hospital, Zhejiang University School of Medicine, Hangzhou, Zhejiang, China

**Keywords:** ALFF, cognitive impairment, COPD, mediation analysis, PAO, precuneus, resting-state fMRI

## Abstract

**Background:**

Cognitive impairment is a common but underrecognized comorbidity in chronic obstructive pulmonary disease (COPD). Lower arterial oxygenation may be associated with altered spontaneous brain activity, but the regional functional correlates linking oxygenation and cognition remain incompletely understood. This study examined amplitude of low-frequency fluctuation (ALFF) abnormalities in COPD patients with cognitive impairment and explored their associations with arterial oxygenation and cognitive performance.

**Methods:**

This cross-sectional study included 90 participants: 30 healthy controls, 30 COPD patients without cognitive impairment, and 30 COPD patients with cognitive impairment. Cognitive status was assessed using the Montreal Cognitive Assessment (MoCA); one point was added for participants with ≤12 years of education, with the adjusted score capped at 30, and an education-adjusted score <26 was used to define cognitive impairment. All participants underwent arterial blood gas analysis and resting-state functional MRI. Whole-brain voxel-wise ALFF differences were evaluated using analysis of covariance, with an uncorrected voxel-wise cluster-forming threshold of *p* < 0.001; clusters were considered significant at a cluster-level FDR-corrected threshold of *p* < 0.05. Adjusted regression, restricted cubic spline, and exploratory cross-sectional mediation analyses were performed in patients with COPD.

**Results:**

COPD patients with cognitive impairment showed lower ALFF in the left precuneus, left putamen, and bilateral parahippocampal gyri, with all clusters surviving cluster-level FDR correction (P_FDR = 0.004–0.021). Higher PaO₂ was associated with higher MoCA total score and higher ALFF in the left precuneus and left putamen after adjustment for multiple comparisons. Higher regional ALFF was also associated with better cognitive performance, with the strongest association observed in the left precuneus. In an exploratory cross-sectional mediation model, left precuneus ALFF statistically accounted for part of the association between PaO₂ and MoCA total score.

**Conclusion:**

Reduced left precuneus ALFF may represent a candidate neuroimaging correlate of lower arterial oxygenation and poorer cognitive performance in COPD. Because of the cross-sectional design, the exploratory mediation findings do not establish temporal or causal relationships and require confirmation in larger longitudinal cohorts.

## Background

Chronic obstructive pulmonary disease (COPD) remains a leading cause of morbidity and mortality worldwide and imposes substantial healthcare costs (1). Population aging and the high prevalence of extrapulmonary comorbidities further amplify this burden (2). Cognitive impairment has emerged as one of the most clinically consequential yet frequently overlooked of these comorbidities. A recent systematic review and meta-analysis estimated that 20%–30% of patients with COPD exhibit cognitive impairment and that approximately 24% meet criteria for mild cognitive impairment (3). Patients with COPD face a substantially elevated risk of cognitive decline compared with individuals without the disease (4). Prevalence can be even higher-reaching up to 77%-among those with concomitant hypoxemia (5).

The functional consequences of cognitive impairment in COPD extend far beyond test scores. Deficits predominantly affect executive function, attention, working memory, and visuospatial abilities (5). These deficits can undermine patients’ capacity for self-management, including correct inhaler technique, adherence to complex medication regimens, participation in pulmonary rehabilitation, and timely recognition of exacerbation symptoms (6). Cognitive impairment is also associated with poorer quality of life and greater functional dependence (7). In patients with severe COPD, selected visuospatial deficits have independently predicted mortality (8). Despite robust evidence of its prevalence and impact, routine cognitive screening has not been integrated into standard respiratory care pathways (7). This represents a major clinical pain point: a prevalent and prognostically important comorbidity remains largely undetected in everyday practice.

The mechanisms linking COPD to cognitive impairment are multifactorial (9). Chronic hypoxemia, reflected in reduced arterial oxygen partial pressure (PaO₂), is widely recognized as a central contributor. Meta-analytic evidence demonstrates a consistent positive correlation between PaO₂ and cognitive performance (10). Hypoxemia promotes oxidative stress, impairs cerebral metabolism, induces neuronal apoptosis, and disrupts neurotransmitter systems. These effects are compounded by systemic inflammation, hypercapnia, vascular dysfunction, and sleep-disordered breathing. Their interplay accelerates structural brain changes and functional network disruption, particularly within the default mode network and fronto-parieto-temporal circuits (11). Resting-state functional MRI (rs-fMRI) provides several complementary approaches for characterizing intrinsic brain function. In the present study, we selected the amplitude of low-frequency fluctuation (ALFF) as the primary imaging metric because it directly quantifies the magnitude of spontaneous low-frequency blood oxygen level-dependent signal fluctuations at the voxel level (12). Unlike seed-based functional connectivity, ALFF does not require selection of an *a priori* reference region and is therefore suitable for whole-brain identification of localized abnormalities in spontaneous neural activity. ALFF is not intended to replace functional connectivity, regional homogeneity, or graph-theoretical measures, which assess different aspects of functional integration and network organization; rather, it was selected because our primary question concerned regional activity amplitude and its associations with arterial oxygenation and cognitive performance. An ALFF study in stable COPD reported reduced spontaneous activity in the posterior cingulate cortex and precuneus (13). Another study localized decreased ALFF to the basal ganglia and thalamus and related these abnormalities to pulmonary function and PaO₂ (14). Static and dynamic ALFF analyses have also identified abnormalities in basal ganglia and hippocampal/parahippocampal regions that correlate with pulmonary or cognitive measures (15). More recent work has identified broader functional-connectivity alterations associated with clinical and cognitive parameters in COPD (16).

Nevertheless, several important gaps remain. Most previous rs-fMRI studies compared patients with COPD as a broad group with healthy controls or primarily examined associations with pulmonary disease severity. Relatively few studies have directly compared COPD patients with and without clinically defined cognitive impairment, making it difficult to distinguish brain functional abnormalities associated with cognitive status from those associated with COPD itself (11). In addition, the continuous and potentially nonlinear relationships between PaO₂ and regional spontaneous brain activity have not been sufficiently characterized. More importantly, it remains unclear whether specific regional ALFF abnormalities are associated with both arterial oxygenation and cognitive performance and whether they statistically account for part of the cross-sectional association between these clinical measures. Addressing these gaps may help identify candidate neuroimaging correlates for future longitudinal and interventional research.

Compared with previous rs-fMRI investigations, the present study combined a three-group design comprising healthy controls, COPD patients without cognitive impairment, and COPD patients with cognitive impairment with whole-brain voxel-wise ALFF analysis, adjusted association models, restricted cubic spline analysis, and exploratory cross-sectional mediation analysis. Previous evidence implicates default mode network dysfunction in COPD (17). ALFF studies have also reported altered spontaneous activity in the precuneus (13). On this basis, we hypothesized that COPD patients with cognitive impairment would show reduced ALFF in default mode and memory-related regions, with the precuneus representing a particularly plausible candidate region. We further hypothesized that lower PaO₂ would be associated with reduced regional ALFF and poorer MoCA performance. The primary imaging analysis remained a whole-brain voxel-wise analysis without restriction to a predefined precuneus region. We subsequently examined whether regional ALFF values, particularly those in the precuneus if identified by the whole-brain analysis, statistically accounted for part of the cross-sectional association between PaO₂ and cognitive performance. Given the cross-sectional design, these analyses were intended to generate mechanistic hypotheses rather than establish temporal or causal relationships.

## Method

### Sample-size considerations

This exploratory neuroimaging study was based on a fixed sample of eligible participants, and no formal *a priori* sample-size calculation for voxel-wise inference was performed. To characterize the detectable effect size of the final sample, a sensitivity power analysis was conducted using G*Power version 3.1.9.7 (18). For an omnibus fixed-effects comparison among three equally sized groups, a total sample size of 90, a two-sided *α* level of 0.05, and 80% power corresponded to a minimum detectable effect size of Cohen’s f* = 0.333. The power to detect an effect size of *f* = 0.40 was 92.6%. This calculation was intended only to provide an approximate indication of power for the omnibus three-group comparison and did not establish adequate power for voxel-wise cluster inference, restricted cubic spline analyses, or mediation analyses. The latter analyses were therefore considered exploratory.

### Study design and participants

This cross-sectional observational study enrolled patients with stable chronic obstructive pulmonary disease (COPD) and healthy controls (HCs). Patients with COPD were consecutively screened from those admitted to the Department of Respiratory Medicine and the intensive care unit of the First Affiliated Hospital, Zhejiang University School of Medicine, between 2021 and 2025.

COPD was diagnosed according to the Global Initiative for Chronic Obstructive Lung Disease criteria, including persistent post-bronchodilator airflow limitation defined as an FEV1/FVC ratio <0.70 in the appropriate clinical context (19). Spirometric airflow-limitation severity was classified according to post-bronchodilator FEV1 percentage predicted as GOLD grade 1 (FEV1 ≥ 80% predicted), grade 2 (50% ≤ FEV1 < 80% predicted), grade 3 (30% ≤ FEV1 < 50% predicted), or grade 4 (FEV1 < 30% predicted) (2). These grades describe the severity of airflow limitation and were not interpreted as a comprehensive measure of overall COPD severity, which also depends on symptoms, exacerbation history, and comorbidities. During the initial eligibility screening, available medical records were reviewed for previously diagnosed obstructive sleep apnea (OSA), prior polysomnography results, and documented use of continuous or bilevel positive airway pressure therapy. Participants with a known clinical diagnosis of OSA were excluded. However, validated OSA screening questionnaires, such as the STOP-BANG questionnaire or Epworth Sleepiness Scale, were not administered systematically, and overnight oximetry or polysomnography was not performed as part of the study protocol. Therefore, previously undiagnosed OSA could not be excluded.

According to cognitive status, patients were classified as COPD without cognitive impairment (COPD-NC) or COPD with cognitive impairment (COPD-CI). HCs were recruited from individuals undergoing routine health examinations or through community-based advertisements during the same study period. All participants underwent standardized clinical assessment, pulmonary function testing, arterial blood gas analysis, cognitive evaluation, and resting-state functional MRI.

### Clinical and cognitive assessments

Global cognitive function was assessed using the Montreal Cognitive Assessment (MoCA), which has a total score ranging from 0 to 30, with higher scores indicating better cognitive performance. In accordance with the original MoCA scoring recommendations, 1 point was added to the raw total score for participants with 12 years of formal education or fewer, with the education-adjusted score capped at 30. An education-adjusted MoCA score <26, corresponding to a score of 0–25, was used as the prespecified screening criterion for cognitive impairment, whereas a score of 26–30 was classified as no cognitive impairment (20). Accordingly, patients with COPD were classified as COPD with cognitive impairment (COPD-CI) or COPD without cognitive impairment (COPD-NC). Because the MoCA is a cognitive screening instrument rather than a stand-alone diagnostic assessment, this threshold was used to identify participants who screened positive for cognitive impairment and not to establish a definitive clinical diagnosis.

Because the MoCA is a cognitive screening instrument rather than a stand-alone diagnostic assessment, cognitive classification was additionally reviewed by trained investigators using the available clinical neuropsychological information. Participants meeting the education-adjusted MoCA criterion and the clinical evaluation criteria were classified as COPD-CI, whereas those not meeting these criteria were classified as COPD-NC.

Pulmonary function parameters included FEV1, FVC, FEV1/FVC ratio, FEV1 percentage predicted, FVC percentage predicted, and GOLD spirometric grade. Arterial blood gas parameters included PaO₂, PaCO₂, SaO₂, and pH. Inflammatory markers included white blood cell count, C-reactive protein, neutrophil-to-lymphocyte ratio, and serum interleukin-6. Domain-specific MoCA scores included visuospatial/executive function, naming, attention, language, abstraction, delayed recall, and orientation. Anxiety symptoms were assessed using the Hamilton Anxiety Rating Scale (21). Depressive symptoms were assessed using the Hamilton Depression Rating Scale (22).

### Resting-state fMRI preprocessing

Resting-state fMRI data were preprocessed in MATLAB R2023b using the Data Processing Assistant for Resting-State fMRI (DPARSF V5.2_210501), which is based on Statistical Parametric Mapping 12 (23). ALFF maps were calculated using REST plus (version 1.27) (24).

A total of 200 functional volumes were acquired for each participant. No initial volumes were discarded before preprocessing; all acquired volumes were entered into the preprocessing pipeline and were subsequently subject to motion-related quality control and scrubbing. The images underwent slice-timing correction followed by six-parameter rigid-body realignment. Each participant’s functional images were co-registered to the corresponding T1-weighted structural image. The structural images were segmented into gray matter, white matter, and cerebrospinal fluid, and the deformation fields derived from segmentation were used to normalize the functional images to Montreal Neurological Institute space. The normalized functional images were resampled to 3 × 3 × 3 mm^3^ isotropic voxels.

Linear trends were removed from the time series. Nuisance regression included the six rigid-body realignment parameters, the mean white-matter signal, and the mean cerebrospinal-fluid signal. Temporal global signal regression was not performed. Volumes with framewise displacement >0.20 mm were identified as motion-contaminated volumes and censored during scrubbing (25). Mean framewise displacement was subsequently included as a covariate in the voxel-wise group analysis.

Normalization of voxel-wise ALFF values by the global mean ALFF value was performed after ALFF calculation and was distinct from temporal global signal regression.

Participants were excluded if head motion exceeded 2.0 mm of translation or 2.0° of rotation in any direction. Framewise displacement was calculated for each participant, and volumes with FD > 0.20 mm were identified as motion-contaminated volumes.

The number and percentage of motion-contaminated and retained volumes were recorded for quality-control comparisons among groups. Mean FD was included as a covariate in the voxel-wise group analysis. Additional head-motion measures, including median and maximum FD, maximum translation, maximum rotation, volumes with FD > 0.20 mm or >0.50 mm, percentage of motion-contaminated volumes, and retained volumes, were compared among groups.

A temporal band of 0.01–0.08 Hz was used to isolate low-frequency BOLD fluctuations. Spatial smoothing was performed using a 6-mm full-width at half-maximum Gaussian kernel.

### ALFF calculation

For each voxel, the preprocessed time series was transformed into the frequency domain using a fast Fourier transform. The square root of the power spectrum was calculated and averaged across the 0.01–0.08 Hz frequency range to obtain the voxel-wise ALFF value (26). To reduce interindividual variability, each voxel’s ALFF value was divided by the global mean ALFF value within the whole-brain mask, yielding a standardized ALFF map. This spatial normalization procedure was distinct from temporal global signal regression.

For regions showing significant group differences in the voxel-wise analysis, mean ALFF values were extracted from the surviving clusters and used for visualization, Bonferroni-corrected *post hoc* comparisons, association analyses with PaO₂ and MoCA total score, restricted cubic spline analyses, and exploratory mediation analyses.

### Voxel-wise ALFF analysis

Whole-brain voxel-wise differences in standardized ALFF values among the HC, COPD-NC, and COPD-CI groups were examined using analysis of covariance (ANCOVA), with age, sex, years of education, body mass index, cumulative smoking exposure, and mean framewise displacement included as covariates. An uncorrected voxel-wise threshold of *p* < 0.001 was used as the primary cluster-forming threshold. Multiple-comparison correction was subsequently performed at the cluster level using the false discovery rate (FDR) procedure, and clusters with a cluster-level FDR-corrected *p* value < 0.05 were considered statistically significant.

For each significant cluster, the cluster size, peak Montreal Neurological Institute coordinates, peak *F* value, and cluster-level FDR-corrected p value were recorded. Mean ALFF values were extracted from the surviving clusters to illustrate the direction of group differences. Pairwise *post hoc* comparisons among the HC, COPD-NC, and COPD-CI groups were performed with Bonferroni correction.

### Regression assumptions and sensitivity analyses

Before interpreting the general linear models, the underlying model assumptions were assessed. Linearity was evaluated using scatterplots and partial-residual plots. Residual distributions were examined using quantile–quantile plots and the Shapiro–Wilk test, and homoscedasticity was evaluated using residual-versus-fitted plots. Multicollinearity was assessed using variance inflation factors, with values <5 considered acceptable. Standardized residuals, leverage values, and Cook’s distance were examined to identify potentially influential observations.

Where departure from a strictly linear association was clinically plausible, restricted cubic spline models were used to characterize the exposure–outcome relationship without imposing a simple linear form (27). Robust-regression sensitivity analyses were additionally performed for the continuous paths in the primary left precuneus model to reduce the influence of potentially influential observations. Covariates were selected *a priori* on clinical and methodological grounds, and smoking status and cumulative smoking exposure were not entered simultaneously into the same model.

### Exploratory cross-sectional mediation analysis

Mediation analyses were conducted solely as exploratory, hypothesis-generating statistical models to evaluate whether regional ALFF statistically accounted for part of the cross-sectional association between PaO₂ and cognitive performance in patients with COPD. PaO₂ was specified as the exposure, regional ALFF as the candidate statistical mediator, and MoCA total score as the outcome.

Separate models were constructed for the left precuneus, left putamen, left parahippocampal gyrus, and right parahippocampal gyrus. The left precuneus was treated as the principal imaging region in the exploratory analyses because it demonstrated the most prominent group difference and strongest association pattern in the preceding whole-brain and regression analyses; the other regions were considered secondary exploratory regions.

The primary mediation model was adjusted for age, sex, education years, body mass index, smoking status, FEV1 percentage predicted, and mean framewise displacement. The framework estimated the PaO₂–ALFF association, the ALFF–MoCA association conditional on PaO₂, the total PaO₂–MoCA association, the residual direct association after accounting for ALFF, and the indirect association through ALFF.

Indirect effects and their 95% confidence intervals were estimated using 5,000 bootstrap resamples (28). The proportion statistically accounted for was calculated as the indirect effect divided by the total effect and was interpreted cautiously.

Because PaO₂, regional ALFF, and MoCA were measured concurrently, temporal ordering could not be established. Cross-sectional mediation analysis may provide biased estimates of longitudinal or causal mediation when the mediator and outcome are assessed at the same time point (29). Moreover, the assumptions of no unmeasured exposure–mediator, mediator–outcome, or exposure–outcome confounding could not be verified. Therefore, the indirect effects and proportions accounted for were interpreted only as statistical decompositions of cross-sectional associations and not as evidence that reduced PaO₂ caused altered regional ALFF or that altered ALFF subsequently caused cognitive impairment.

## Results

**Figure 1:**
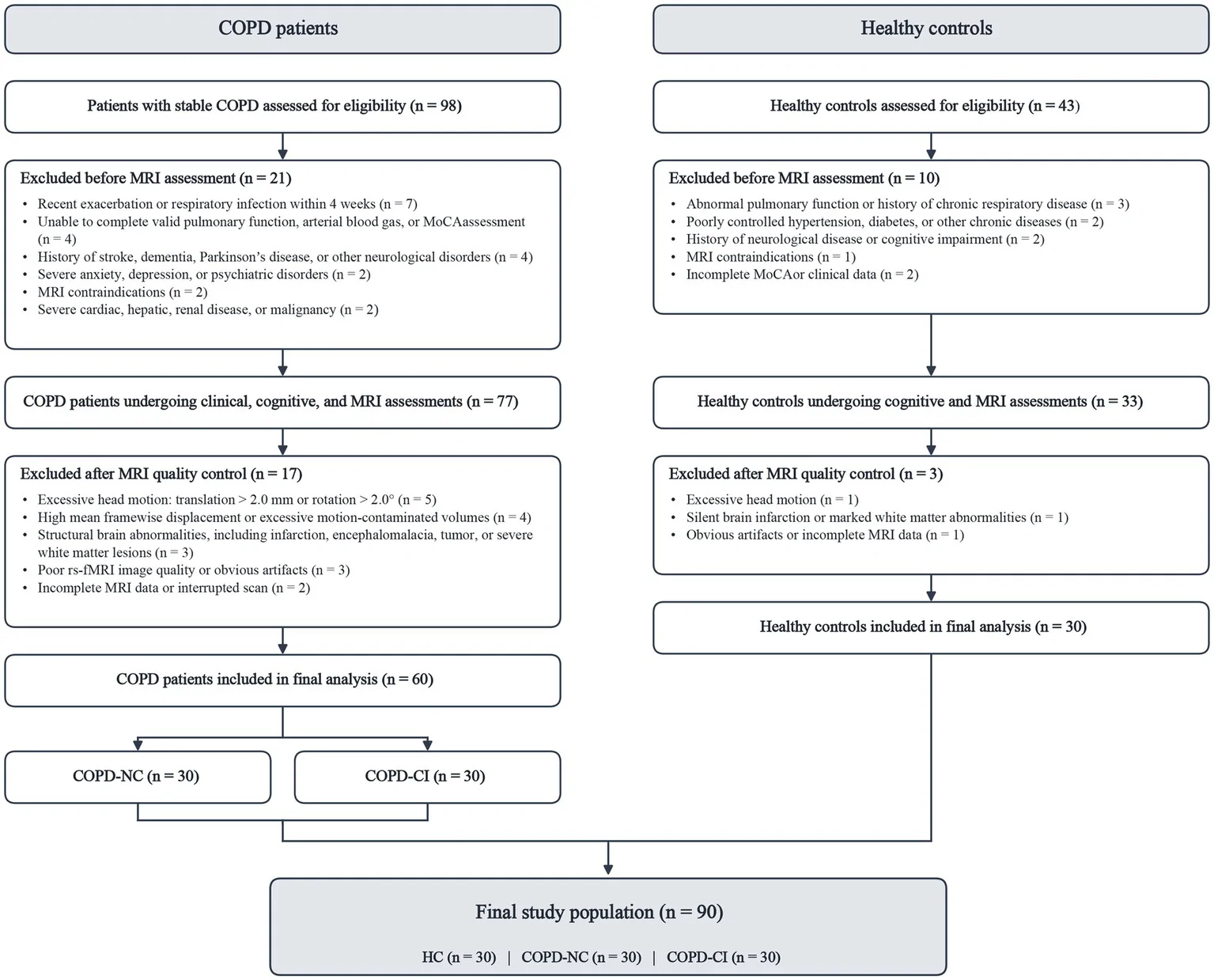
Flowchart of participant selection. A total of 98 patients with stable COPD and 43 healthy controls were assessed for eligibility. After clinical screening and MRI quality control, 60 COPD patients and 30 healthy controls were included in the final analysis. The final cohort consisted of 30 healthy controls, 30 COPD patients without cognitive impairment, and 30 COPD patients with cognitive impairment.

The study flowchart is shown in Figure 1. Of 98 patients with stable COPD and 43 healthy controls assessed for eligibility, 60 patients with COPD and 30 healthy controls were included in the final analysis. The study population comprised 30 healthy controls, 30 patients with COPD without cognitive impairment (COPD-NC), and 30 patients with COPD with cognitive impairment (COPD-CI).

Demographic and general clinical characteristics are summarized in Table 1. Most demographic variables, vascular comorbidities, anxiety and depression scores, and head-motion measures did not differ significantly among the groups. Smoking status differed among groups (*p* = 0.018; Cramér’s V = 0.329), and cumulative smoking exposure was greater in the COPD groups than in healthy controls (*p* = 0.026; ε^2^ = 0.061).

**Table 1:** Demographic and general clinical characteristics among the three groups.

Variable	HC (*n* = 30)	COPD-NC (*n* = 30)	COPD-CI (*n* = 30)	*p* value	Effect size
Age, years	63.2 ± 6.5	64.8 ± 7.1	66.5 ± 7.3	0.173	η^2^ = 0.040
Male sex, *n* (%)	18 (60.0)	22 (73.3)	23 (76.7)	0.351	*V* = 0.157
Education, years	11.7 ± 3.0	10.9 ± 3.2	10.1 ± 3.4	0.156	η^2^ = 0.042
BMI, kg/m^2^	23.9 ± 2.7	23.2 ± 3.0	22.6 ± 3.1	0.214	η^2^ = 0.035
Current or former smoker, *n* (%)	12 (40.0)	21 (70.0)	23 (76.7)	0.018	*V* = 0.329
Smoking exposure, pack-years	14.6 ± 18.2	31.8 ± 22.5	35.4 ± 24.1	0.026	ε^2^ = 0.061
Alcohol use, *n* (%)	8 (26.7)	10 (33.3)	12 (40.0)	0.556	*V* = 0.115
Hypertension, *n* (%)	9 (30.0)	11 (36.7)	13 (43.3)	0.577	*V* = 0.113
Diabetes mellitus, *n* (%)	4 (13.3)	5 (16.7)	7 (23.3)	0.594	*V* = 0.109
Coronary artery disease, *n* (%)	3 (10.0)	5 (16.7)	6 (20.0)	0.553	*V* = 0.115
History of TIA, *n* (%)	0 (0.0)	1 (3.3)	2 (6.7)	0.356	*V* = 0.152
HAMA score	5.1 ± 3.2	6.4 ± 3.7	7.5 ± 4.1	0.067	η^2^ = 0.060
HAMD score	5.4 ± 3.5	6.7 ± 3.8	7.8 ± 4.3	0.083	η^2^ = 0.056
Mean FD, mm	0.086 ± 0.031	0.092 ± 0.035	0.101 ± 0.039	0.286	η^2^ = 0.028
Maximum translation, mm	0.84 ± 0.31	0.91 ± 0.34	0.97 ± 0.38	0.337	η^2^ = 0.025
Maximum rotation, degrees	0.72 ± 0.29	0.76 ± 0.32	0.82 ± 0.35	0.491	η^2^ = 0.016

Data are presented as mean ± standard deviation or *n* (%). Group differences were assessed using one-way ANOVA, Kruskal–Wallis test, *χ*^2^ test, or Fisher’s exact test, as appropriate. Effect sizes are presented as η^2^ for continuous variables analyzed by one-way ANOVA, ε^2^ for smoking exposure analyzed by the Kruskal–Wallis test, and Cramér’s V for categorical variables. Baseline descriptive variables were not subjected to multiple-comparison adjustment, and statistical nonsignificance was not interpreted as proof of group equivalence. FD, framewise displacement; HC, healthy controls; COPD-NC, COPD patients without cognitive impairment; COPD-CI, COPD patients with cognitive impairment; HAMA, Hamilton Anxiety Rating Scale; HAMD, Hamilton Depression Rating Scale; TIA, transient ischemic attack.

Among the 60 patients with COPD, 26 (43.3%) had GOLD grade 2 airflow limitation, 25 (41.7%) had grade 3, and 9 (15.0%) had grade 4. GOLD-grade distribution did not differ significantly between the COPD-NC and COPD-CI groups (*p* = 0.229).

Pulmonary and clinical characteristics are presented in Table 2. Compared with the COPD-NC group, the COPD-CI group had lower PaO₂ (68.9 ± 9.4 vs. 75.6 ± 8.7 mmHg; mean difference, −6.7 mmHg; 95% CI, −11.4 to −2.0; Hedges’ g = −0.73; P_FDR = 0.016) and lower SaO₂ (92.3 ± 3.0% vs. 94.2 ± 2.1%; mean difference, −1.9 percentage points; 95% CI, −3.24 to −0.56; Hedges’ g = −0.72; P_FDR = 0.016). CRP, neutrophil-to-lymphocyte ratio, CAT score, mMRC score, and previous-year exacerbations differed at the nominal level but did not remain significant after within-domain FDR correction. No other pulmonary-function, inflammatory, or treatment-related variable differed significantly after FDR correction.

**Table 2:** Pulmonary function, arterial blood gas parameters, inflammatory markers, and COPD burden in COPD-NC and COPD-CI patients.

Variable	COPD-NC (*n* = 30)	COPD-CI (*n* = 30)	Effect estimate (95% CI where applicable)	Effect size (95% CI)	*p* value	*P*_FDR_
Pulmonary function						
FEV_1_, L	1.62 ± 0.48	1.43 ± 0.44	Mean difference −0.19 (−0.43 to 0.05)	Hedges’ *g* − 0.41 (−0.91 to 0.10)	0.116	0.194
FEV_1_, % predicted	56.8 ± 14.7	49.9 ± 13.6	Mean difference −6.9 (−14.2 to 0.4)	Hedges’ *g* − 0.48 (−0.99 to 0.03)	0.065	0.194
FVC, L	2.91 ± 0.72	2.68 ± 0.69	Mean difference −0.23 (−0.59 to 0.13)	Hedges’ *g* − 0.32 (−0.82 to 0.18)	0.213	0.229
FVC, % predicted	72.4 ± 14.0	66.9 ± 13.7	Mean difference −5.5 (−12.7 to 1.7)	Hedges’ *g* − 0.39 (−0.90 to 0.11)	0.129	0.194
FEV_1_/FVC	0.52 ± 0.09	0.48 ± 0.10	Mean difference −0.04 (−0.09 to 0.01)	Hedges’ *g* − 0.42 (−0.92 to 0.09)	0.110	0.194
GOLD spirometric grade, *n* (%)			—	Cramér’s *V* 0.214	0.229	0.229
II	16 (53.3)	10 (33.3)				
III	11 (36.7)	14 (46.7)				
IV	3 (10.0)	6 (20.0)				
Arterial blood gas parameters						
PaO_2_, mmHg	75.6 ± 8.7	68.9 ± 9.4	Mean difference −6.7 (−11.4 to −2.0)	Hedges’ *g* − 0.73 (−1.25 to −0.21)	**0.006**	**0.016**
PaCO_2_, mmHg	42.1 ± 5.8	45.3 ± 6.7	Mean difference 3.2 (−0.04 to 6.44)	Hedges’ *g* 0.50 (−0.01 to 1.01)	0.053	0.071
SaO_2_, %	94.2 ± 2.1	92.3 ± 3.0	Mean difference −1.9 (−3.24 to −0.56)	Hedges’ *g* − 0.72 (−1.24 to −0.21)	**0.008**	**0.016**
pH	7.41 ± 0.03	7.40 ± 0.04	Mean difference −0.01 (−0.03 to 0.01)	Hedges’ *g* − 0.28 (−0.78 to 0.22)	0.287	0.287
Inflammatory markers						
WBC, ×10^9^/L	6.4 ± 1.7	7.0 ± 2.0	Mean difference 0.6 (−0.36 to 1.56)	Hedges’ *g* 0.32 (−0.18 to 0.82)	0.220	0.220
CRP, mg/L	3.8 (2.1, 6.5)	5.9 (3.2, 9.4)	Median difference 2.1	Rank-based *r* 0.268 (0.015 to 0.489)	0.038	0.072
NLR	2.3 (1.8, 3.2)	3.1 (2.1, 4.5)	Median difference 0.8	Rank-based *r* 0.258 (0.004 to 0.480)	0.046	0.072
IL-6, pg./mL	4.9 (3.2, 7.1)	6.7 (4.1, 9.6)	Median difference 1.8	Rank-based *r* 0.249 (−0.006 to 0.473)	0.054	0.072
COPD burden and treatment-related variables						
Disease duration, years	7.6 ± 4.1	8.9 ± 4.8	Mean difference 1.3 (−1.0 to 3.6)	Hedges’ *g* 0.29 (−0.21 to 0.79)	0.267	0.401
CAT score	15.2 ± 5.4	18.8 ± 6.1	Mean difference 3.6 (0.62 to 6.58)	Hedges’ *g* 0.62 (0.10 to 1.13)	0.019	0.082
mMRC dyspnea score	1.6 ± 0.7	2.0 ± 0.8	Mean difference 0.4 (0.01 to 0.79)	Hedges’ *g* 0.53 (0.02 to 1.03)	0.041	0.082
Acute exacerbations in previous year, *n*	1.0 (0.0, 2.0)	2.0 (1.0, 3.0)	Median difference 1.0	Rank-based *r* 0.275 (0.023 to 0.495)	0.033	0.082
Long-term oxygen therapy, *n* (%)	5 (16.7)	8 (26.7)	OR 1.82 (0.52 to 6.38)	Cramér’s *V* 0.121	0.347	0.416
ICS-containing inhaled therapy, *n* (%)	12 (40.0)	15 (50.0)	OR 1.50 (0.54 to 4.17)	Cramér’s *V* 0.101	0.436	0.436

Data are presented as mean ± standard deviation, median (interquartile range), or *n* (%). Effect estimates for continuous variables are expressed as the COPD-CI minus COPD-NC mean or median difference; binary categorical estimates are odds ratios. Effect sizes are Hedges’ g for normally distributed continuous variables, rank-based r for Mann–Whitney U comparisons, and Cramér’s V for categorical variables. P_FDR_ values were calculated using the Benjamini–Hochberg procedure within each prespecified clinical domain. A positive effect estimate indicates a higher value in COPD-CI, and a negative estimate indicates a lower value in COPD-CI. COPD-NC, COPD without cognitive impairment; COPD-CI, COPD with cognitive impairment; FEV_1_, forced expiratory volume in 1 s; FVC, forced vital capacity; GOLD, Global Initiative for Chronic Obstructive Lung Disease; PaO_2_, arterial partial pressure of oxygen; PaCO_2_, arterial partial pressure of carbon dioxide; SaO_2_, arterial oxygen saturation; WBC, white blood cell count; CRP, C-reactive protein; NLR, neutrophil-to-lymphocyte ratio; IL-6, interleukin-6; CAT, COPD Assessment Test; mMRC, modified Medical Research Council; ICS, inhaled corticosteroid; CI, confidence interval. There is statistical significance for bold values.

### Cognitive performance

Global and domain-specific cognitive performance is presented in Table 3. MoCA total score differed among the three groups (F[2,87] = 110.21, *p* < 0.001, η^2^ = 0.717; 95% CI, 0.609–0.778). The COPD-CI group had lower scores than both the HC and COPD-NC groups (both P_Bonferroni < 0.001), whereas the HC and COPD-NC groups did not differ (P_Bonferroni = 1.000). Because the education-adjusted MoCA score contributed to cognitive-group classification, this total-score difference should be interpreted descriptively.

**Table 3:** Global and domain-specific cognitive performance among the three groups.

Variable	HC (*n* = 30)	COPD-NC (*n* = 30)	COPD-CI (*n* = 30)	Omnibus test statistic	Effect size (95% CI)	Nominal *P*	*P*_FDR_	Bonferroni-adjusted pairwise comparisons
MoCA total score (0–30)	27.1 ± 0.9	26.9 ± 1.2	22.8 ± 1.6	*F*(2,87) = 110.21	η^2^ = 0.717 (0.609–0.778)	**<0.001**	—	HC vs. COPD-NC: 1.000 HC vs. COPD-CI: <0.001 COPD-NC vs. COPD-CI: <0.001
Visuospatial/executive (0–5)	4.6 ± 0.7	4.5 ± 0.8	3.6 ± 1.0	*F*(2,87) = 12.82	η^2^ = 0.228 (0.081–0.357)	**<0.001**	**<0.001**	HC vs. COPD-NC: 1.000 HC vs. COPD-CI: <0.001 COPD-NC vs. COPD-CI: <0.001
Naming (0–3)	2.8 ± 0.4	2.7 ± 0.5	2.6 ± 0.5	*F*(2,87) = 1.36	η^2^ = 0.030 (0.000–0.114)	0.261	0.261	HC vs. COPD-NC: 1.000 HC vs. COPD-CI: 0.307 COPD-NC vs. COPD-CI: 1.000
Attention (0–6)	5.5 ± 0.7	5.6 ± 0.9	4.5 ± 1.0	*F*(2,87) = 14.48	η^2^ = 0.250 (0.098–0.379)	**<0.001**	**<0.001**	HC vs. COPD-NC: 1.000 HC vs. COPD-CI: <0.001 COPD-NC vs. COPD-CI: <0.001
Language (0–3)	2.7 ± 0.5	2.6 ± 0.6	2.3 ± 0.7	*F*(2,87) = 3.55	η^2^ = 0.075 (0.000–0.184)	**0.033**	**0.046**	HC vs. COPD-NC: 1.000 HC vs. COPD-CI: 0.037 COPD-NC vs. COPD-CI: 0.175
Abstraction (0–2)	1.8 ± 0.4	1.7 ± 0.5	1.4 ± 0.5	*F*(2,87) = 5.91	η^2^ = 0.120 (0.014–0.241)	**0.004**	**0.007**	HC vs. COPD-NC: 1.000 HC vs. COPD-CI: 0.004 COPD-NC vs. COPD-CI: 0.046
Delayed recall (0–5)	4.2 ± 0.8	3.9 ± 1.0	2.8 ± 1.2	*F*(2,87) = 15.88	η^2^ = 0.267 (0.112–0.396)	**<0.001**	**<0.001**	HC vs. COPD-NC: 0.764 HC vs. COPD-CI: <0.001 COPD-NC vs. COPD-CI: <0.001
Orientation (0–6)	5.7 ± 0.5	5.9 ± 0.6	5.6 ± 0.6	*F*(2,87) = 2.16	η^2^ = 0.047 (0.000–0.144)	0.121	0.141	HC vs. COPD-NC: 0.530 HC vs. COPD-CI: 1.000 COPD-NC vs. COPD-CI: 0.132

Data are presented as mean ± standard deviation. Omnibus group differences were assessed using one-way analysis of variance. Effect sizes are reported as eta squared (η^2^) with approximate 95% confidence intervals derived from the noncentral F distribution. The MoCA total score was the prespecified primary cognitive outcome and was not included in the multiplicity adjustment. P_FDR_ values for the seven domain-specific scores were calculated using the Benjamini–Hochberg procedure. Pairwise comparisons were based on the pooled ANOVA error term and adjusted for the three comparisons within each outcome using the Bonferroni method. COPD-CI was defined using education-adjusted MoCA criteria and clinical neuropsychological evaluation; therefore, the MoCA total-score group difference is partly inherent to the classification procedure and should be interpreted descriptively. MoCA, Montreal Cognitive Assessment; HC, healthy controls; COPD-NC, COPD without cognitive impairment; COPD-CI, COPD with cognitive impairment; CI, confidence interval; FDR, false discovery rate. There is statistical significance for bold values.

After FDR correction across the seven cognitive domains, significant group effects remained for visuospatial/executive function, attention, language, abstraction, and delayed recall, whereas naming and orientation did not differ significantly. The COPD-CI group had lower visuospatial/executive, attention, and delayed-recall scores than both comparison groups. Language was lower in COPD-CI than in HC, while abstraction was lower in COPD-CI than in both HC and COPD-NC. The largest domain-level effects were observed for delayed recall, attention, and visuospatial/executive function.

Head-motion quality-control results are shown in Supplementary Table 1. Mean, median, and maximum framewise displacement; maximum translation and rotation; the numbers of volumes exceeding FD thresholds of 0.20 and 0.50 mm; the percentage of motion-contaminated volumes; and the number of unflagged volumes did not differ significantly among the three groups. All included participants met the predefined head-motion criteria.

**Figure 2:**
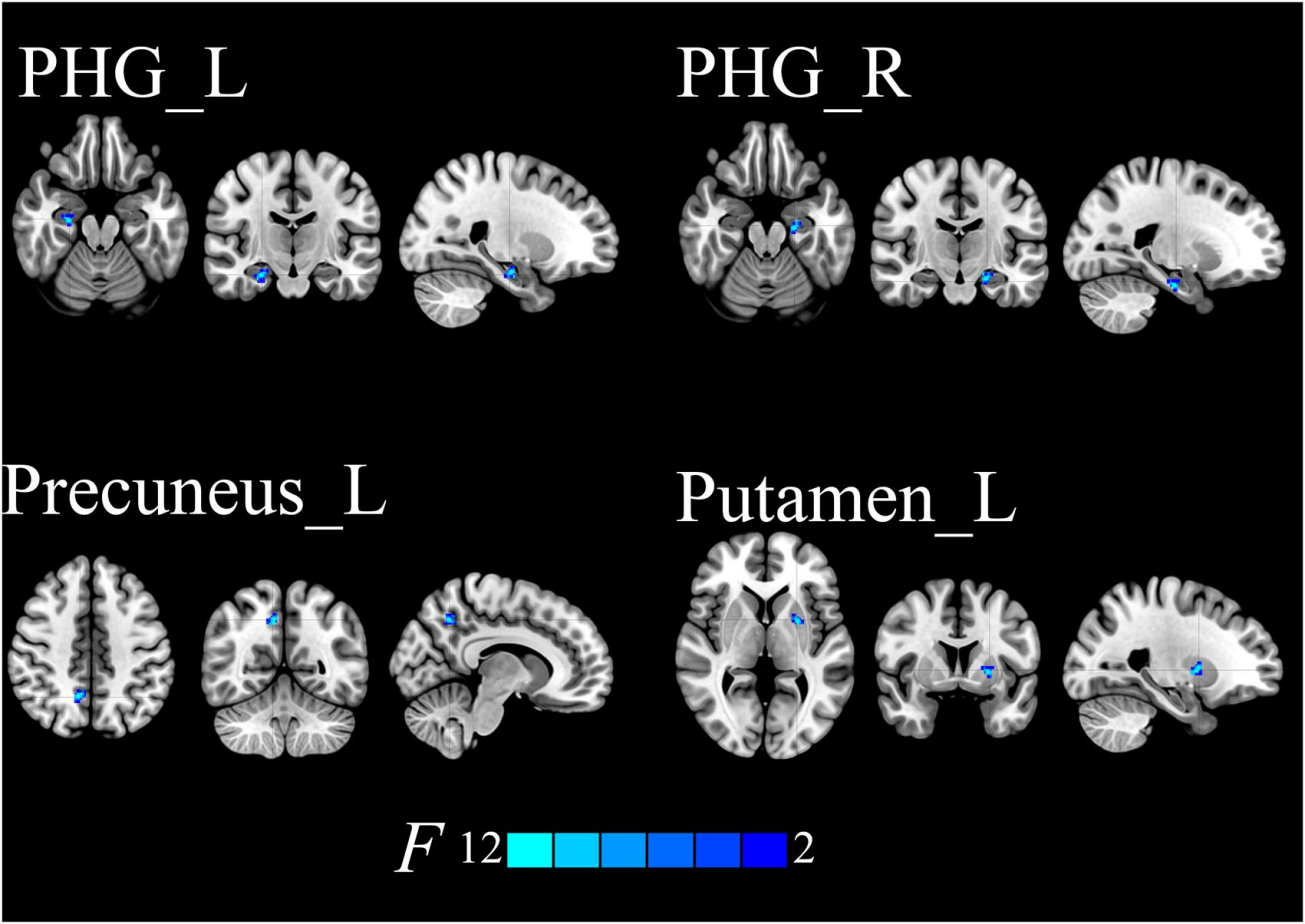
Brain regions showing significant ALFF differences among groups. Voxel-wise ANCOVA identified significant group differences in the left parahippocampal gyrus (PHG_L), right parahippocampal gyrus (PHG_R), left precuneus, and left putamen. The statistical maps are overlaid on standard brain templates and displayed in axial, coronal, and sagittal views. The color bar represents *F* values from voxel-wise ANCOVA. Directionality of group differences is shown by extracted ALFF values and Bonferroni-corrected *post hoc* comparisons. Statistical maps were generated using an uncorrected voxel-wise cluster-forming threshold of *p* < 0.001, followed by cluster-level FDR correction at *P*_FDR_ < 0.05.

Voxel-wise ANCOVA identified significant group effects in the left precuneus (peak *F* = 10.42, P_FDR = 0.004), left putamen (peak *F* = 9.36, P_FDR = 0.007), left parahippocampal gyrus (peak *F* = 8.27, P_FDR = 0.014), and right parahippocampal gyrus (peak *F* = 7.82, P_FDR = 0.021; Table 4; Figure 2).

**Table 4:** Brain regions showing significant ALFF differences among HC, COPD-NC, and COPD-CI groups.

Brain region	Hemisphere	Cluster size (voxels)	Peak MNI coordinates (*x*, *y*, *z*)	Peak *F* value	Cluster-level *P*_FDR_	Bonferroni-adjusted *post hoc* comparisons
Precuneus	Left	86	−6, −60, 42	10.42	0.004	COPD-CI < HC, *P*_Bonferroni_ = 0.003 COPD-CI < COPD-NC, *P*_Bonferroni_ = 0.041
Putamen	Left	64	−24, 6, 0	9.36	0.007	COPD-CI < HC, *P*_Bonferroni_ = 0.006 COPD-CI < COPD-NC, *P*_Bonferroni_ = 0.047
Parahippocampal gyrus	Left	51	−27, −33, −12	8.27	0.014	COPD-CI < HC, *P*_Bonferroni_ = 0.012 COPD-CI < COPD-NC, *P*_Bonferroni_ = 0.048
Parahippocampal gyrus	Right	47	30, −30, −15	7.82	0.021	COPD-CI < HC, *P*_Bonferroni_ = 0.018 COPD-CI vs. COPD-NC, *P*_Bonferroni_ = 0.052

Whole-brain group differences were assessed using voxel-wise analysis of covariance (ANCOVA), with age, sex, years of education, body mass index, smoking exposure, and mean framewise displacement included as covariates. Clusters were initially defined using an uncorrected voxel-wise cluster-forming threshold of *p* < 0.001 and were considered statistically significant at a cluster-level false discovery rate (FDR)-corrected threshold of *p*_FDR_< 0.05. The *P* < sub>FDR</sub> values reported in the sixth column represent cluster-level corrected *p* values. Pairwise post hoc comparisons were performed using mean ALFF values extracted from each significant cluster, with Bonferroni adjustment for the three possible between-group comparisons within each cluster. Only comparisons involving the COPD-CI group are displayed because these were the prespecified contrasts of primary interest; all *p* values in the final column are Bonferroni-adjusted. The difference in right parahippocampal ALFF between the COPD-CI and COPD-NC groups did not reach the Bonferroni-corrected significance threshold. ALFF, amplitude of low-frequency fluctuation; HC, healthy controls; COPD-NC, patients with COPD without cognitive impairment; COPD-CI, patients with COPD with cognitive impairment; MNI, Montreal Neurological Institute; FDR, false discovery rate.

Bonferroni-adjusted comparisons showed lower ALFF in the COPD-CI group than in healthy controls in all four regions (P_Bonferroni = 0.003–0.018). Compared with COPD-NC, COPD-CI showed lower ALFF in the left precuneus (P_Bonferroni = 0.041), left putamen (P_Bonferroni = 0.047), and left parahippocampal gyrus (P_Bonferroni = 0.048). The right parahippocampal difference between the two COPD groups did not reach statistical significance (P_Bonferroni = 0.052). Overall, ALFF was lowest in the COPD-CI group across all four clusters.

**Figure 3:**
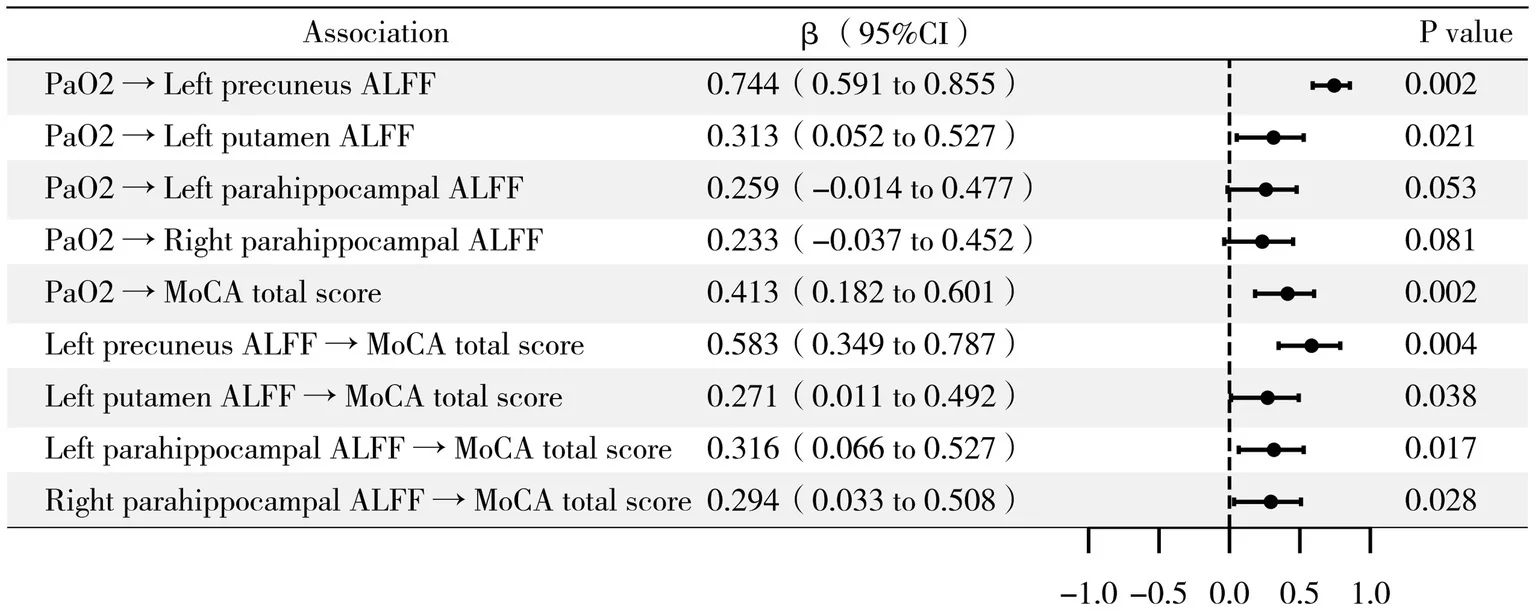
Associations among PaO₂, regional ALFF values, and MoCA total score in COPD patients. Forest plot showing standardized regression coefficients and 95% confidence intervals for the associations among PaO₂, regional ALFF values, and MoCA total score. PaO₂ was positively associated with MoCA total score and with ALFF values in the left precuneus and left putamen. Regional ALFF values in the left precuneus, left putamen, and bilateral parahippocampal gyri were positively associated with MoCA total score.

Adjusted associations are presented in Figure 3. Higher PaO₂ was associated with a higher MoCA total score (standardized *β* = 0.413; 95% CI, 0.159–0.668; *p* = 0.002).

After FDR correction, PaO₂ was positively associated with ALFF in the left precuneus (β = 0.604; 95% CI, 0.257–0.951; P_FDR = 0.004) and left putamen (*β* = 0.313; 95% CI, 0.049–0.577; P_FDR = 0.042), but not in the bilateral parahippocampal gyri.

Higher ALFF was associated with higher MoCA total score in the left precuneus (*β* = 0.483; 95% CI, 0.161–0.805; P_FDR = 0.016), left parahippocampal gyrus (*β* = 0.316; 95% CI, 0.059–0.573; P_FDR = 0.034), right parahippocampal gyrus (*β* = 0.294; 95% CI, 0.033–0.555; P_FDR = 0.037), and left putamen (*β* = 0.271; 95% CI, 0.016–0.526; P_FDR = 0.038).

Restricted cubic spline analyses are shown in Supplementary Figure 2. PaO₂ was associated with MoCA total score (P_overall = 0.004), with weak evidence of nonlinearity (P_nonlinearity = 0.047). PaO₂ was also associated with left precuneus ALFF (P_overall = 0.006), without significant evidence of nonlinearity (P_nonlinearity = 0.104). The association between PaO₂ and the odds of COPD-CI was significant overall (P_overall = 0.006), while the nonlinearity test did not reach statistical significance (P_nonlinearity = 0.058).

**Figure 4:**
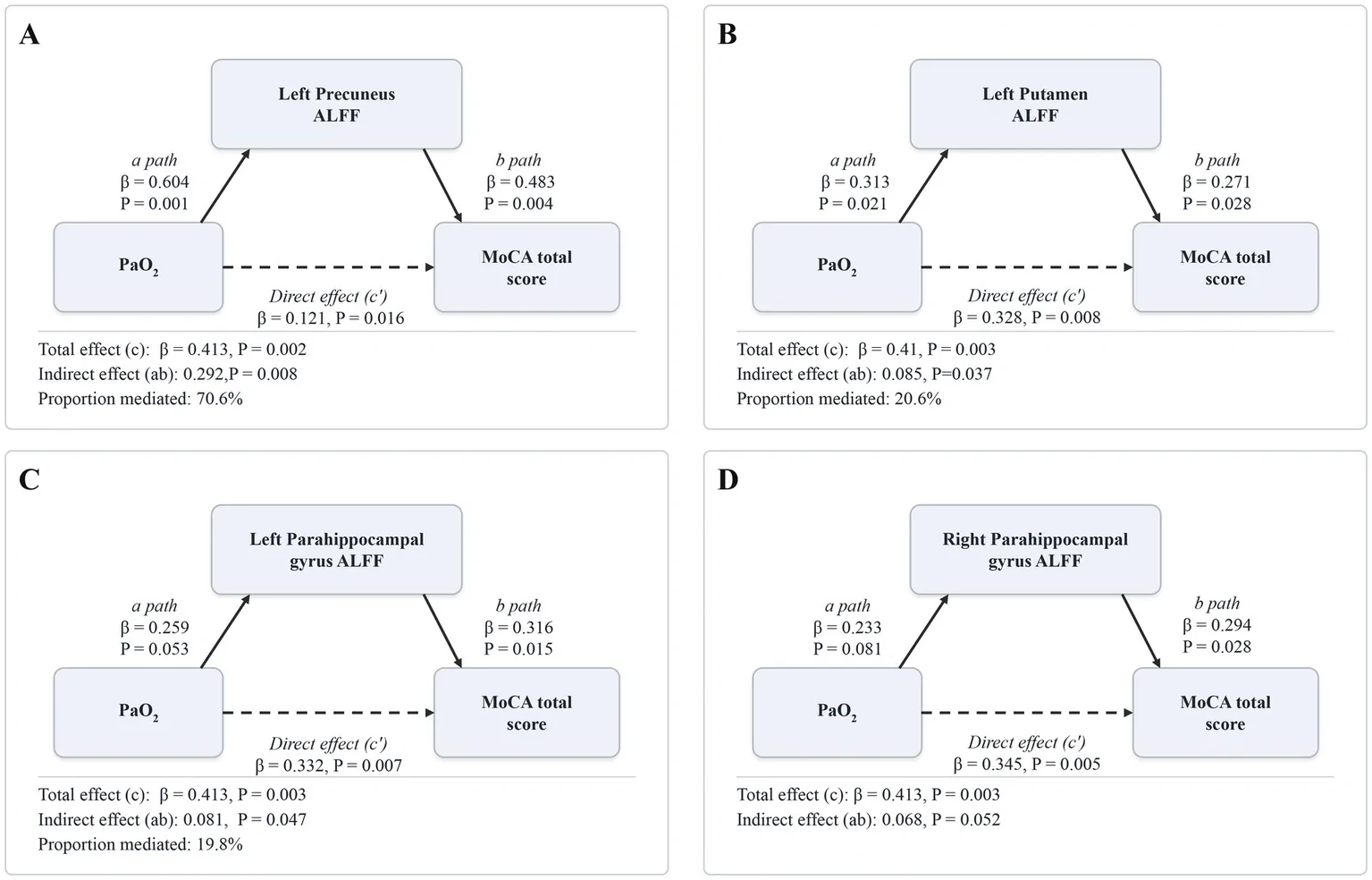
Mediation analyses of regional ALFF in the association between PaO₂ and MoCA total score. Mediation models were constructed with PaO₂ as the independent variable, MoCA total score as the dependent variable, and regional ALFF values as mediators. Four brain regions were evaluated separately: left precuneus ALFF **(A)**, left putamen ALFF **(B)**, left parahippocampal gyrus ALFF **(C)**, and right parahippocampal gyrus ALFF **(D)**. The left precuneus showed the largest indirect association, with an estimated proportion statistically accounted for of 70.6%. Because PaO₂, ALFF, and MoCA were measured concurrently, this percentage should not be interpreted as a causal mediated proportion and may overestimate any corresponding longitudinal indirect effect. The left putamen and left parahippocampal gyrus showed smaller statistically significant indirect associations, whereas the indirect association through the right parahippocampal gyrus did not reach statistical significance.

Exploratory cross-sectional mediation models are shown in Figure 4. In the primary left precuneus model, the total association between PaO₂ and MoCA total score was 0.413, the residual direct association was 0.121, and the indirect association through left precuneus ALFF was 0.292 (*p* = 0.008), corresponding to an estimated proportion statistically accounted for of 70.6%.

Smaller indirect associations were observed through the left putamen (0.085, *p* = 0.037) and left parahippocampal gyrus (0.081, *p* = 0.047), whereas the indirect association through the right parahippocampal gyrus did not reach statistical significance (0.068, *p* = 0.052). Because all variables were assessed concurrently, these findings represent exploratory statistical decompositions of cross-sectional associations rather than evidence of temporal or causal mediation.

## Discussion

In this cross-sectional study, COPD patients with cognitive impairment showed reduced regional spontaneous brain activity involving the left precuneus, left putamen, and bilateral parahippocampal gyri. Among patients with COPD, lower arterial oxygenation was associated with lower ALFF in the left precuneus and left putamen, while regional ALFF values were associated with cognitive performance. The left precuneus demonstrated the most consistent pattern across the group, regression, spline, and exploratory indirect-association analyses. These findings identify a candidate regional functional signature of oxygenation-related cognitive vulnerability in COPD, but they do not establish temporal sequence or causality.

Our findings are broadly consistent with earlier ALFF studies in COPD. Wang et al. reported lower ALFF in the posterior cingulate cortex and precuneus in stable COPD, with regional activity related to respiratory physiological measures (13). Lu et al. (14) subsequently identified altered basal-ganglia ALFF associated with pulmonary ventilation indices. Studies examining static and dynamic local neural activity have also reported abnormalities in the basal ganglia and parahippocampal/hippocampal regions, with associations with pulmonary and cognitive measures (15). The present study extends this literature by directly separating COPD patients according to cognitive status rather than treating COPD as a single group. The intermediate ALFF values observed in COPD-NC patients are compatible with a graded cross-sectional pattern, although they should not be interpreted as evidence that these patients will subsequently progress to cognitive impairment.

The regional findings should also be considered within the broader multimodal neuroimaging literature. Default-mode-network studies have demonstrated altered connectivity involving posterior cingulate, hippocampal, and parahippocampal regions in COPD, with relationships to cognitive performance and disease severity (17). Degree-centrality and seed-based connectivity analyses have identified disrupted communication involving the precuneus, putamen, cingulate, insular, and frontoparietal regions (30). Static and dynamic network analyses similarly suggest that COPD is associated with altered interactions across the default mode, executive-control, visual, and sensorimotor networks (31). Structural MRI studies have reported gray-matter and network-level abnormalities related to attention, language, and global cognitive performance (32). Combined arterial-spin-labeling and resting-state fMRI studies have identified altered cerebral blood flow and neurovascular coupling in regions associated with MoCA and domain-specific scores (33). Taken together, these studies suggest that COPD-related cognitive impairment is unlikely to reflect dysfunction of a single brain region. Rather, the current pattern may represent local manifestations of broader disruption involving posterior default-mode, medial-temporal, frontostriatal, vascular, and white-matter systems.

The involvement of the precuneus is biologically plausible because this region participates in the integration of internally directed cognition, visuospatial processing, attention, and memory-related network activity (34). Reduced precuneus ALFF is therefore consistent with the prominent visuospatial/executive, attention, and delayed-recall abnormalities observed in the COPD-CI group. The parahippocampal gyri form part of medial-temporal systems involved in contextual and memory processing, whereas the putamen participates in frontostriatal circuits relevant to cognitive control, motor planning, and executive function. Nevertheless, regional functional specialization should not be overstated, because ALFF reflects the amplitude of local BOLD fluctuations and does not directly measure neuronal firing, synaptic integrity, or effective connectivity (35).

Lower PaO₂ may be one contributor to the observed functional abnormalities, but it is unlikely to be the only explanation. Recurrent or sustained reductions in oxygen availability may interact with hypercapnia, cerebrovascular dysfunction, impaired neurovascular coupling, smoking exposure, systemic inflammation, sleep-disordered breathing, reduced physical activity, and comorbid vascular disease (9). In the present study, CRP and neutrophil-to-lymphocyte ratio differed between the COPD groups at the nominal level but did not remain significant after within-domain FDR correction. The data therefore do not provide firm evidence that systemic inflammation independently explains the imaging findings. Similarly, the spline analyses suggest that the association with PaO₂ may extend across the observed oxygenation range, but the modest sample size and borderline evidence of nonlinearity preclude identification of a reliable physiological threshold.

The mediation analysis requires particularly cautious interpretation. Because PaO₂, regional ALFF, and MoCA were measured concurrently, temporal ordering could not be established, and the indirect associations cannot determine whether lower oxygenation preceded altered regional brain activity or whether altered ALFF preceded cognitive impairment. Cross-sectional mediation estimates can differ substantially from the corresponding longitudinal processes and may indicate an indirect effect even when no longitudinal mediation exists (29). Accordingly, the estimated 70.6% proportion statistically accounted for by left precuneus ALFF should not be interpreted as the proportion of a causal effect mediated through the precuneus. Given the unusually large magnitude of this estimate for a complex cognitive phenotype, it likely overestimates any corresponding longitudinal or causal mediated proportion. The estimate may have been inflated by the concurrent measurement of the exposure, candidate mediator, and outcome; residual or unmeasured confounding; the modest sample size; measurement variability; and selection of the regional ALFF measure from clusters identified within the same dataset. Therefore, the 70.6% estimate should be regarded only as an exploratory statistical decomposition of cross-sectional associations rather than a precise or causal mediation estimate. Longitudinal studies with repeated measurements of oxygenation, brain function, and cognition, together with more comprehensive control of potential confounders, are required to evaluate whether a temporal indirect pathway exists.

The findings nevertheless have several potential clinical implications. Cognitive assessment may be particularly relevant in COPD patients with lower PaO₂, difficulty following treatment instructions, repeated inhaler errors, poor adherence, frequent exacerbations, or limited engagement with rehabilitation. Cognitive impairment can interfere with medication management, symptom monitoring, decision-making, and inhaler use (6). An abnormal screening result should prompt a more comprehensive clinical assessment rather than be interpreted in isolation. In practice, cognitively vulnerable patients may benefit from simplified instructions, repeated demonstration of inhaler technique, teach-back methods, written or visual reminders, caregiver involvement, and closer follow-up.

Cognitive impairment should not automatically exclude patients from pulmonary rehabilitation. COPD patients with mild cognitive impairment can still achieve clinically meaningful improvements in health status and exercise capacity after rehabilitation (36). Cognitive deficits may, however, create barriers to treatment participation and indicate a need for more individualized support (37). Preliminary randomized evidence also suggests that adding structured cognitive training to pulmonary rehabilitation may improve selected cognitive outcomes, although these findings require confirmation in larger and more diverse populations (38). The present study does not demonstrate that oxygen supplementation, pulmonary rehabilitation, or cognitive training will normalize ALFF or prevent cognitive decline. It instead provides a rationale for future trials to include cognition and brain-functional outcomes alongside conventional respiratory and exercise outcomes.

At present, regional ALFF should remain a research-level measure. The overlap between groups, dependence on acquisition and preprocessing choices, modest sample size, and absence of external validation preclude its use as a diagnostic biomarker or as a basis for individualized oxygen prescriptions. Future studies could evaluate whether combining clinical variables, nocturnal oxygenation, arterial blood gases, neuropsychological profiles, structural MRI, perfusion imaging, and functional-network measures improves prediction beyond standard clinical assessment. Prospective studies should also determine whether regional brain-function abnormalities are reversible, stable, or progressive and whether changes are associated with treatment response.

This study has several strengths. The three-group design allowed brain-functional abnormalities associated with cognitive status to be examined separately from those broadly associated with COPD. Whole-brain voxel-wise ALFF analysis avoided restriction to a single predefined seed, while cluster-level FDR correction and Bonferroni-adjusted *post hoc* testing reduced the likelihood of false-positive regional findings. MRI was acquired using a standardized scanner and protocol, and extensive head-motion indices were examined, with mean framewise displacement included as a covariate. Cognitive classification incorporated education adjustment and trained clinical assessment. The analysis also integrated covariate-adjusted regression, multiple-comparison correction, flexible spline modeling, and sensitivity analyses. These features improve the internal consistency of the observed associations, although they do not overcome the limitations of the cross-sectional design.

Several limitations should be acknowledged. First, the cross-sectional, single-center design prevents assessment of temporal relationships and limits generalizability. Second, the sample size of 30 participants per group limits the precision of the spline, mediation, and sensitivity estimates. The equal-sized groups were useful for comparison but cannot be used to estimate the prevalence of cognitive impairment in the source COPD population. Third, because MoCA contributed to the definition of COPD-CI, the between-group difference in MoCA total score is partly inherent to the classification procedure. Fourth, a single daytime PaO₂ measurement may not reflect cumulative hypoxemic burden, nocturnal desaturation, exercise-related desaturation, or longitudinal oxygen exposure. Although available medical records were reviewed and participants with a previously documented diagnosis of OSA were excluded, validated OSA screening questionnaires, overnight oximetry, and polysomnography were not systematically performed. Consequently, undiagnosed OSA and COPD–OSA overlap syndrome could not be excluded and may have contributed to nocturnal intermittent hypoxemia, altered brain function, and cognitive impairment. Residual confounding by sleep-disordered breathing therefore remains possible. Fifth, unmeasured medication, vascular, lifestyle, nutritional, and inflammatory factors may have affected both brain function and cognitive performance. Sixth, regional values were extracted from clusters identified in the same sample, and the regional associations therefore require independent replication. Finally, ALFF measures local spontaneous BOLD amplitude but does not characterize structural injury, cerebral perfusion, dynamic connectivity, network topology, or directionality of information flow. Multimodal and longitudinal studies are needed to integrate these complementary aspects.

In conclusion, reduced left precuneus ALFF may represent a candidate neuroimaging correlate of lower arterial oxygenation and poorer cognitive performance in COPD. The findings support further evaluation of cognitive vulnerability as part of comprehensive COPD research and care, while prospective longitudinal and interventional studies are required to determine temporal relationships, reproducibility, reversibility, and clinical utility.

## Data Availability

The raw data supporting the conclusions of this article will be made available by the authors, without undue reservation.

## Supplementary material

SUPPLEMENTARY FIGURE 1 Extracted regional ALFF values among the three groups. Box-and-scatter plots showing mean ALFF values in the left precuneus **(A)**, left putamen **(B)**, left parahippocampal gyrus **(C)**, and right parahippocampal gyrus **(D)** among healthy controls, COPD patients without cognitive impairment, and COPD patients with cognitive impairment. COPD patients with cognitive impairment showed lower ALFF values in these regions, with the most prominent differences observed in the left precuneus and left putamen.

SUPPLEMENTARY FIGURE 2 Restricted cubic spline analyses of the associations between PaO₂ and cognitive/neuroimaging outcomes in COPD patients. Restricted cubic spline curves showing the associations of PaO₂ with adjusted MoCA total score **(A)**, adjusted left precuneus ALFF **(B)**, and the odds of COPD-related cognitive impairment **(C)**. PaO₂ showed significant overall associations with MoCA total score, left precuneus ALFF, and COPD-CI risk. P-overall indicates the overall association, and P-nonlinearity indicates evidence for deviation from linearity.

## References

[1] Wang Z Lin J Liang L Huang F Yao X Peng K . Global, regional, and national burden of chronic obstructive pulmonary disease and its attributable risk factors from 1990 to 2021: an analysis for the global burden of disease study 2021. Respir Res. (2025) 26:2. doi: 10.1186/s12931-024-03051-2 39748260 PMC11697803

[2] Barnes PJ Celli BR. Systemic manifestations and comorbidities of COPD. Eur Respir J. (2009) 33:1165–85. doi: 10.1183/09031936.00128008, 19407051

[3] Zhang Z Yang P Xiao G Li B He M Yang Y . Prevalence and risk factors of cognitive impairment in COPD: a systematic review and meta-analysis. Public Health Nurs. (2025) 42:1389–407. doi: 10.1111/phn.13524 39794894

[4] Wang J Li X Lei S Zhang D Zhang S Zhang H . Risk of dementia or cognitive impairment in COPD patients: a meta-analysis of cohort studies. Front Aging Neurosci. (2022) 14:20220909. doi: 10.3389/fnagi.2022.962562, 36158542 PMC9500359

[5] Torres-Sánchez I Rodríguez-Alzueta E Cabrera-Martos I López-Torres I Moreno-Ramírez MP Valenza MC. Cognitive impairment in COPD: a systematic review. J Bras Pneumol. (2015) 41:182–90. doi: 10.1590/s1806-37132015000004424 25909154 PMC4428856

[6] Baird C Lovell J Johnson M Shiell K Ibrahim JE. The impact of cognitive impairment on self-management in chronic obstructive pulmonary disease: a systematic review. Respir Med. (2017) 129:130–9. doi: 10.1016/j.rmed.2017.06.006, 28732820

[7] Siraj RA. Comorbid cognitive impairment in chronic obstructive pulmonary disease (COPD): current understanding, risk factors, implications for clinical practice, and suggested interventions. Medicina (Kaunas). (2023) 59:732. doi: 10.3390/medicina59040732, 37109690 PMC10146750

[8] Antonelli-Incalzi R Corsonello A Pedone C Trojano L Acanfora D Spada A . Drawing impairment predicts mortality in severe COPD. Chest. (2006) 130:1687–94. doi: 10.1378/chest.130.6.1687, 17166983

[9] Panda S Walsh M Anghel I Ghosh AJ. Mechanisms and risk factors of cognitive impairment in COPD. CHEST Pulm. (2026) 4:100196. doi: 10.1016/j.chpulm.2025.100196, 41919092 PMC13035366

[10] Wen XH Li Y Han D Sun L Ren PX Ren D. The relationship between cognitive function and arterial partial pressure O2 in patients with COPD: a meta-analysis. Medicine (Baltimore). (2018) 97:e9599. doi: 10.1097/md.0000000000009599, 29369175 PMC5794359

[11] He JK Han XY Tan YS Yao Z Dong Y Feng C. Aberrant brain functional network in COPD patients with cognitive impairment: clinical manifestations, mechanisms and therapeutic strategies. J Neuroinflammation. (2025) 23:30. doi: 10.1186/s12974-025-03651-9 41422064 PMC12836923

[12] Zou QH Zhu CZ Yang Y Zuo XN Long XY Cao QJ . An improved approach to detection of amplitude of low-frequency fluctuation (ALFF) for resting-state fMRI: fractional ALFF. J Neurosci Methods. (2008) 172:137–41. 20080422. doi: 10.1016/j.jneumeth.2008.04.012 18501969 PMC3902859

[13] Wang W Li H Peng D Luo J Xin H Yu H . Abnormal intrinsic brain activities in stable patients with COPD: a resting-state functional MRI study. Neuropsychiatr Dis Treat. (2018) 14:2763–72. doi: 10.2147/ndt.S180325 30425494 PMC6200435

[14] Lu CQ Xu W Zeng CH Ge LY Wang YC Meng XP . Altered amplitude of low-frequency fluctuation in basal ganglia correlates to pulmonary ventilation function in COPD patients: a resting-state fMRI study. Brain Behav. (2019) 9:e01336. doi: 10.1002/brb3.1336 31140760 PMC6625472

[15] Lv Z Chen Q Jiang Y Hu P Zhang L Bai T . Abnormal static and dynamic local-neural activity in COPD and its relationship with pulmonary function and cognitive impairments. Front Hum Neurosci. (2020) 14:580238. doi: 10.3389/fnhum.2020.580238 33519397 PMC7843446

[16] Han KI Yeo Y Jo HJ Jo MJ Park Y Park TS . Abnormal brain functional connectivity in patients with chronic obstructive pulmonary disease and correlations with clinical and cognitive parameters. Int J Chron Obstruct Pulmon Dis. (2025) 20:971–85. doi: 10.2147/copd.S505271, 40207024 PMC11980928

[17] Hu X Wang H Tu Y Fei M Yin M Fei G . Alterations of the default mode network and cognitive impairments in patients with chronic obstructive pulmonary disease. Int J Chron Obstruct Pulmon Dis. (2018) 13:519–28. doi: 10.2147/copd.S146870, 29445270 PMC5808710

[18] Faul F Erdfelder E Buchner A Lang AG. Statistical power analyses using G*Power 3.1: tests for correlation and regression analyses. Behav Res Methods. (2009) 41:1149–60. doi: 10.3758/brm.41.4.1149, 19897823

[19] Agustí A Celli BR Criner GJ Halpin D Anzueto A Barnes P . Global initiative for chronic obstructive lung disease 2023 report: GOLD executive summary. Am J Respir Crit Care Med. (2023) 207:819–37. doi: 10.1164/rccm.202301-0106PP, 36856433 PMC10111975

[20] Nasreddine ZS Phillips NA Bédirian V Charbonneau S Whitehead V Collin I . The Montreal cognitive assessment, MoCA: a brief screening tool for mild cognitive impairment. J Am Geriatr Soc. (2005) 53:695–9. doi: 10.1111/j.1532-5415.2005.53221.x, 15817019

[21] Hamilton M. The assessment of anxiety states by rating. Br J Med Psychol. (1959) 32:50–5. doi: 10.1111/j.2044-8341.1959.tb00467.x, 13638508

[22] Hamilton M. A rating scale for depression. J Neurol Neurosurg Psychiatry. (1960) 23:56–62. doi: 10.1136/jnnp.23.1.56, 14399272 PMC495331

[23] Chao-Gan Y Yu-Feng Z. DPARSF: a MATLAB toolbox for "pipeline" data analysis of resting-state fMRI. Front Syst Neurosci. (2010) 4:13. doi: 10.3389/fnsys.2010.00013 20577591 PMC2889691

[24] Jia XZ Wang J Sun HY Zhang H Liao W Wang Z . RESTplus: an improved toolkit for resting-state functional magnetic resonance imaging data processing. Sci Bull (Beijing). (2019) 64:953–4. 20190513. doi: 10.1016/j.scib.2019.05.008 36659803

[25] Power JD Mitra A Laumann TO Snyder AZ Schlaggar BL Petersen SE. Methods to detect, characterize, and remove motion artifact in resting state fMRI. NeuroImage. (2014) 84:320–41. 20130829. doi: 10.1016/j.neuroimage.2013.08.048 23994314 PMC3849338

[26] Zang YF He Y Zhu CZ Cao QJ Sui MQ Liang M . Altered baseline brain activity in children with ADHD revealed by resting-state functional MRI. Brain Dev. (2007) 29:83–91. 20060817. doi: 10.1016/j.braindev.2006.07.002 16919409

[27] Desquilbet L Mariotti F. Dose-response analyses using restricted cubic spline functions in public health research. Stat Med. (2010) 29:1037–57. 20100119. doi: 10.1002/sim.3841 20087875

[28] Preacher KJ Hayes AF. Asymptotic and resampling strategies for assessing and comparing indirect effects in multiple mediator models. Behav Res Methods. (2008) 40:879–91. doi: 10.3758/brm.40.3.879, 18697684

[29] Maxwell SE Cole DA. Bias in cross-sectional analyses of longitudinal mediation. Psychol Methods. (2007) 12:23–44. doi: 10.1037/1082-989x.12.1.23, 17402810

[30] Li H Xin H Yu J Yu H Zhang J Wang W . Abnormal intrinsic functional hubs and connectivity in stable patients with COPD: a resting-state MRI study. Brain Imaging Behav. (2020) 14:573–85. doi: 10.1007/s11682-019-00130-7, 31187474 PMC7160072

[31] Tang F Li L Peng D Yu J Xin H Tang X . Abnormal static and dynamic functional network connectivity in stable chronic obstructive pulmonary disease. Front Aging Neurosci. (2022) 14:20221017. doi: 10.3389/fnagi.2022.1009232, 36325191 PMC9618865

[32] Wang W Wang P Li Q Peng Z Wang X Wang G . Alterations of grey matter volumes and network-level functions in patients with stable chronic obstructive pulmonary disease. Neurosci Lett. (2020) 720:134748. doi: 10.1016/j.neulet.2020.134748, 31935432

[33] Peng Z Zhang HT Wang G Zhang J Qian S Zhao Y . Cerebral neurovascular alterations in stable chronic obstructive pulmonary disease: a preliminary fMRI study. PeerJ. (2022) 10:e14249. doi: 10.7717/peerj.14249, 36405017 PMC9671032

[34] Cavanna AE Trimble MR. The precuneus: a review of its functional anatomy and behavioural correlates. Brain. (2006) 129:564–83. 20060106. doi: 10.1093/brain/awl004 16399806

[35] Logothetis NK. What we can do and what we cannot do with fMRI. Nature. (2008) 453:869–78. doi: 10.1038/nature06976, 18548064

[36] Andrianopoulos V Gloeckl R Schneeberger T Jarosch I Vogiatzis I Hume E . Benefits of pulmonary rehabilitation in COPD patients with mild cognitive impairment - a pilot study. Respir Med. (2021) 185:20210523. doi: 10.1016/j.rmed.2021.106478, 34038843

[37] Andrianopoulos V Gloeckl R Vogiatzis I Kenn K. Cognitive impairment in COPD: should cognitive evaluation be part of respiratory assessment? Breathe (Sheff). (2017) 13:e1–9. doi: 10.1183/20734735.001417, 29184593 PMC5702891

[38] Tabka O Sanaa I Mekki M Acheche A Paillard T Trabelsi Y. Effect of a pulmonary rehabilitation program combined with cognitive training on exercise tolerance and cognitive functions among Tunisian male patients with chronic obstructive pulmonary disease: a randomized controlled trial. Chron Respir Dis. (2023) 20:14799731231201643. doi: 10.1177/14799731231201643, 37691169 PMC10494516

