# Supplementary material for: Left precuneus ALFF is associated with arterial oxygenation and cognitive impairment in patients with COPD: a resting-state fMRI study
Source: Front Neurol. 2026 Aug 19;17:1906087. doi: 10.3389/fneur.2026.1906087 (PMC13533676; doi:10.3389/fneur.2026.1906087)
Supplement: Supplementary file 1 [file Table_1.DOCX]

**Supplementary Table 1. Head motion quality control among the three groups**

| **Variable** | **HC (n = 30)** | **COPD-NC**  **(n = 30)** | **COPD-CI**  **(n = 30)** | **P value** |
| --- | --- | --- | --- | --- |
| Mean FD, mm | 0.086 ± 0.031 | 0.092 ± 0.035 | 0.101 ± 0.039 | 0.286 |
| Median FD, mm | 0.079 (0.064-0.103) | 0.085 (0.066-0.112) | 0.094 (0.071-0.121) | 0.241 |
| Maximum FD, mm | 0.42 ± 0.17 | 0.46 ± 0.19 | 0.51 ± 0.22 | 0.258 |
| Maximum translation, mm | 0.84 ± 0.31 | 0.91 ± 0.34 | 0.97 ± 0.38 | 0.337 |
| Maximum rotation, degrees | 0.72 ± 0.29 | 0.76 ± 0.32 | 0.82 ± 0.35 | 0.491 |
| Volumes with FD > 0.20 mm, n | 8.1 ± 5.2 | 9.6 ± 6.1 | 11.4 ± 7.0 | 0.161 |
| Volumes with FD > 0.50 mm, n | 0.6 ± 1.0 | 0.8 ± 1.2 | 1.1 ± 1.5 | 0.312 |
| Scrubbed volumes, % | 4.1 ± 2.6 | 4.8 ± 3.1 | 5.7 ± 3.5 | 0.146 |
| Retained volumes after scrubbing, n | 191.9 ± 5.2 | 190.4 ± 6.1 | 188.6 ± 7.0 | 0.149 |
| Participants with mean FD > 0.15 mm, n (%) | 2 (6.7) | 3 (10.0) | 4 (13.3) | 0.674 |

Data are presented as mean ± standard deviation, median (interquartile range), or n (%). Group differences were assessed using one-way ANOVA, Kruskal-Wallis test, or χ²/Fisher’s exact test, as appropriate. FD, framewise displacement; HC, healthy controls; COPD-NC, COPD patients without cognitive impairment; COPD-CI, COPD patients with cognitive impairment. All participants included in the final ALFF analysis met the predefined head motion criteria. Mean FD was included as a covariate in all voxel-wise ALFF analyses**.**
