# Supplementary material for: Left precuneus ALFF is associated with arterial oxygenation and cognitive impairment in patients with COPD: a resting-state fMRI study
Source: Front Neurol. 2026 Aug 19;17:1906087. doi: 10.3389/fneur.2026.1906087 (PMC13533676; doi:10.3389/fneur.2026.1906087)
Supplement: Supplementary file 2 [file Table_2.DOCX]

**Supplementary Table 2. Sensitivity analyses of the associations among PaO₂, left precuneus ALFF, and MoCA score**

| **Sensitivity analysis** | **n** | **PaO₂ → MoCA B (95% CI), P** | **PaO₂ → left precuneus ALFF B (95% CI), P** | **Left precuneus ALFF → MoCA B (95% CI), P** | **Indirect effect ab (bootstrap 95% CI), P** | **Interpretation** |
| --- | --- | --- | --- | --- | --- | --- |
| Primary model | 60 | 0.119 (0.044 to 0.194), P = 0.003 | 0.0049 (0.0013 to 0.0085), P = 0.011 | 4.24 (0.59 to 7.89), P = 0.024 | 0.021 (0.004 to 0.045), P = 0.022 | Partial mediation supported |
| Further adjusted for PaCO₂ and CRP | 60 | 0.108 (0.029 to 0.187), P = 0.008 | 0.0045 (0.0008 to 0.0082), P = 0.019 | 3.89 (0.21 to 7.57), P = 0.038 | 0.018 (0.003 to 0.041), P = 0.030 | Direction and magnitude largely unchanged |
| Further adjusted for HAMA and HAMD scores | 60 | 0.103 (0.023 to 0.183), P = 0.012 | 0.0047 (0.0009 to 0.0085), P = 0.016 | 3.65 (0.01 to 7.29), P = 0.049 | 0.017 (0.002 to 0.039), P = 0.042 | Association not fully explained by mood symptoms |
| Further adjusted for CAT score and mMRC grade | 60 | 0.096 (0.014 to 0.178), P = 0.021 | 0.0043 (0.0004 to 0.0082), P = 0.032 | 3.52 (-0.12 to 7.16), P = 0.058 | 0.015 (-0.001 to 0.037), P = 0.061 | Effect attenuated but remained directionally consistent |
| Excluding participants with relatively high mean FD (>0.15 mm) | 55 | 0.113 (0.034 to 0.192), P = 0.006 | 0.0046 (0.0008 to 0.0084), P = 0.018 | 4.05 (0.42 to 7.68), P = 0.031 | 0.019 (0.003 to 0.043), P = 0.028 | Findings unlikely to be driven by head motion |
| Excluding patients receiving regular oxygen therapy | 52 | 0.104 (0.022 to 0.186), P = 0.014 | 0.0042 (0.0003 to 0.0081), P = 0.036 | 3.74 (0.06 to 7.42), P = 0.047 | 0.016 (0.002 to 0.039), P = 0.044 | Results remained comparable after removing oxygen-treated patients |
| Robust regression for continuous paths | 60 | 0.111 (0.033 to 0.189), P = 0.006 | 0.0044 (0.0007 to 0.0081), P = 0.021 | 3.91 (0.30 to 7.52), P = 0.035 | 0.017 (0.002 to 0.040), P = 0.039 | Similar pattern after reducing influence of outliers |

The primary mediation model included PaO₂ as the exposure, left precuneus ALFF as the mediator, and MoCA total score as the outcome. The primary model was adjusted for age, sex, education years, BMI, smoking status, FEV1% predicted, and mean framewise displacement.

Sensitivity models additionally adjusted for PaCO₂ and CRP, mood scores, or COPD symptom burden, or excluded participants with relatively high head motion or regular oxygen therapy. Indirect effects were estimated using bootstrap resampling.
