# Supplementary material for: Left precuneus ALFF is associated with arterial oxygenation and cognitive impairment in patients with COPD: a resting-state fMRI study
Source: Front Neurol. 2026 Aug 19;17:1906087. doi: 10.3389/fneur.2026.1906087 (PMC13533676; doi:10.3389/fneur.2026.1906087)
Supplement: Supplementary file 3 [file Table_3.DOCX]

**Supplementary Table 3. Brain regions showing exploratory ALFF differences before FDR correction**

| **Brain region** | **Hemisphere** | **Cluster size**  **(voxels)** | **Peak MNI coordinates**  **(x, y, z)** | **Peak F value** | **Uncorrected P value** | **FDR status** | **HC**  **(n=30)** | **COPD-NC**  **(n=30)** | **COPD-CI**  **(n=30)** | **Post hoc comparison** |
| --- | --- | --- | --- | --- | --- | --- | --- | --- | --- | --- |
| Precuneus | Left | 112 | −6, −60, 42 | 10.42 | 0.0007 | Survived FDR | 0.58 ± 0.20 | 0.43 ± 0.21 | 0.26 ± 0.19 | COPD-CI < HC, P=0.003; COPD-CI < COPD-NC, P=0.041 |
| Putamen | Left | 91 | −24, 6, 0 | 9.36 | 0.0013 | Survived FDR | 0.51 ± 0.18 | 0.37 ± 0.20 | 0.20 ± 0.17 | COPD-CI < HC, P=0.006; COPD-CI < COPD-NC, P=0.047 |
| Parahippocampal gyrus | Left | 74 | −27, −33, −12 | 8.27 | 0.0028 | Survived FDR | 0.46 ± 0.19 | 0.34 ± 0.18 | 0.19 ± 0.17 | COPD-CI < HC, P=0.012; COPD-CI < COPD-NC, P=0.048 |
| Parahippocampal gyrus | Right | 69 | 30, −30, −15 | 7.82 | 0.0046 | Survived FDR | 0.44 ± 0.20 | 0.32 ± 0.18 | 0.18 ± 0.16 | COPD-CI < HC, P=0.018; COPD-CI < COPD-NC, P=0.052 |
| Middle frontal gyrus | Left | 58 | −36, 36, 24 | 7.21 | 0.0068 | Not FDR-significant | 0.49 ± 0.19 | 0.42 ± 0.21 | 0.27 ± 0.18 | COPD-CI < HC, P=0.021; COPD-CI < COPD-NC, P=0.073 |
| Inferior parietal lobule / Angular gyrus | Right | 52 | 45, −60, 33 | 6.84 | 0.0094 | Not FDR-significant | 0.47 ± 0.20 | 0.39 ± 0.19 | 0.28 ± 0.18 | COPD-CI < HC, P=0.028; COPD-CI < COPD-NC, P=0.084 |
| Anterior cingulate cortex | Bilateral/medial | 46 | 0, 36, 18 | 6.52 | 0.0127 | Not FDR-significant | 0.45 ± 0.18 | 0.37 ± 0.20 | 0.26 ± 0.17 | COPD-CI < HC, P=0.034; COPD-CI < COPD-NC, P=0.091 |
| Superior temporal gyrus | Right | 43 | 54, −18, 0 | 6.18 | 0.0165 | Not FDR-significant | 0.43 ± 0.19 | 0.34 ± 0.19 | 0.27 ± 0.18 | COPD-CI < HC, P=0.041; COPD-CI < COPD-NC, P=0.102 |
| Thalamus | Left | 39 | −12, −18, 9 | 5.92 | 0.0209 | Not FDR-significant | 0.42 ± 0.18 | 0.36 ± 0.19 | 0.27 ± 0.17 | COPD-CI < HC, P=0.047; COPD-CI < COPD-NC, P=0.118 |
| Insula | Right | 36 | 36, 12, −3 | 5.68 | 0.0248 | Not FDR-significant | 0.41 ± 0.18 | 0.35 ± 0.19 | 0.26 ± 0.17 | COPD-CI < HC, P=0.056; COPD-CI < COPD-NC, P=0.132 |
| Cerebellum Crus I | Left | 34 | −30, −72, −30 | 5.41 | 0.0306 | Not FDR-significant | 0.39 ± 0.17 | 0.33 ± 0.18 | 0.25 ± 0.16 | COPD-CI < HC, P=0.064; COPD-CI < COPD-NC, P=0.149 |
| Cerebellum lobule VI | Right | 31 | 24, −63, −24 | 5.17 | 0.0369 | Not FDR-significant | 0.38 ± 0.18 | 0.34 ± 0.18 | 0.24 ± 0.16 | COPD-CI < HC, P=0.078; COPD-CI < COPD-NC, P=0.166 |

Group differences were examined using ANCOVA with age, sex, education years, BMI, smoking exposure, and mean framewise displacement as covariates. The first four clusters are consistent with regions that survived FDR correction in the main Table 4; all other regions are exploratory uncorrected findings and should not be interpreted as confirmatory results. Regional ALFF values are presented as mean ± SD extracted from the corresponding clusters. ALFF, amplitude of low-frequency fluctuation; FDR, false discovery rate; HC, healthy controls; COPD-NC, COPD patients without cognitive impairment; COPD-CI, COPD patients with cognitive impairment; MNI, Montreal Neurological Institute.
